# Modulation of bacterial cell size and growth rate via activation of a cell envelope stress response

**DOI:** 10.1128/mbio.02281-25

**Published:** 2025-09-22

**Authors:** Amanda Miguel, Matylda Zietek, Handuo Shi, Anna Sueki, Federico Corona, Lisa Maier, Jolanda Verheul, Tanneke den Blaauwen, David Van Valen, Athanasios Typas, Kerwyn Casey Huang

**Affiliations:** 1 Department of Bioengineering, Stanford University 171702, Stanford, California, USA; 2 Genome Biology Unit, EMBL Heidelberg 9471 , Heidelberg, Germany; 3 Department of Microbiology and Immunology, Stanford University School of Medicine 10624 , Stanford, California, USA; 4 Faculty of Biosciences, Collaboration for joint PhD degree between EMBL and Heidelberg University 9144, Heidelberg, Germany; 5 Faculty of Natural Sciences, Mathematics, and Computer Science, Swammerdam Institute for Life Sciences, University of Amsterdam 1234, Amsterdam, the Netherlands; 6 Department of Biology, California Institute of Technology 6469, Pasadena, California, USA; 7 Molecular Systems Biology Unit, EMBL Heidelberg 9471 , Heidelberg, Germany; 8 Chan Zuckerberg Biohub 578083, San Francisco, California, USA; University of California, Berkeley, Berkeley, California, USA

**Keywords:** Rcs phosphorelay, cell shape, FtsZ, cell division, growth rate, A22

## Abstract

Cells mount elaborate responses to environmental stresses across multiple scales. In enterobacteria, the Rcs phosphorelay is activated by cell-envelope damage and by alterations to periplasmic thickness and cell width. To explore Rcs activation in the absence of external stresses*,* we investigated the physiological and morphological consequences of Rcs activation in *Escherichia coli*. We used an inducible variant of the lipoprotein RcsF that mislocalizes to the inner membrane (RcsF^IM^) and constitutively activates the Rcs system. RcsF^IM^ expression slowed growth and reduced the cell length added per cell cycle, independent of Rcs-regulated capsule production. Concurrently, the concentration of the division protein FtsZ increased, and division rings formed closer together in filamentous cells. Depletion of the Rcs negative regulator IgaA also slowed growth and elevated FtsZ levels, indicating that IgaA is essential due to growth inhibition in its absence. In contrast, treatment with the Rcs-activating antibiotic A22 did not affect growth rate or FtsZ concentration and restored normal growth in cells in which the Rcs system was pre-induced with RcsF^IM^ expression. Hence, the cellular response to A22 buffers the Rcs-dependent effect on cell growth. Together, our findings suggest that Rcs modulates cell division indirectly through growth-rate changes and that activation of the same pathway in different contexts can produce distinct physiological outcomes.

**IMPORTANCE:**

Bacteria must coordinate their growth rate, shape, and division to survive and flourish, yet how these cellular properties are maintained in the face of environmental stresses is poorly understood. Working with *Escherichia coli*, we show that activating the Rcs phosphorelay, an envelope stress-signaling system, in the absence of external stresses slows growth, shortens cells, and increases the concentration of the key division protein FtsZ, leading to more closely spaced division sites. Depleting the levels of IgaA, a regulator of the Rcs pathway, yielded similar phenotypes. However, activating Rcs via drug-induced cell-wall disruption did not affect growth rate, indicating that the physiological impact of this pathway depends on the context of activation. Our findings reveal links among cell growth, shape homeostasis, and cell envelope stress. Understanding this coupling further will provide new avenues to predict and modulate bacterial growth and physiology during stress.

## INTRODUCTION

The environment plays a key role in determining the physiological and physical state of a bacterial cell. In natural conditions, bacteria are exposed to varied and often stressful environments that can include changes to pH, temperature, and nutrient availability, as well as the presence of harmful chemicals such as antibiotics. The myriad ways that bacteria respond to such environmental changes include modulation of growth rate, protein composition, cell envelope modification, and cell size and shape (1, 2). Many of these changes in behavior are governed by complex signaling pathways, each with its own set of stimuli. These stimuli can overlap; for example, as *Escherichia coli* cells encounter starvation, they express the master transcriptional regulator RpoS, which activates programs involved in osmotic, oxidative, and envelope stress (3, 4). Due to the overlap of these stress responses, it remains an open question as to how stress response pathways specifically alter cellular physiology.

The Rcs phosphorelay responds to damage in the cell envelope of Gram-negative bacteria (5, 6). In a chemical genomics screen of the *E. coli* Keio knockout library, deletion of genes in the Rcs pathway resulted in sensitivity to cell shape-perturbing drugs such as A22 and mecillinam (7), suggesting that the Rcs system may play a yet unidentified role in helping cells adjust to shape changes. The Rcs system senses envelope stress via the outer membrane lipoprotein RcsF. Under normal growth conditions, RcsF is transported to the outer membrane and is ultimately surface exposed (8, 9). However, when its transport to the outer membrane or surface exposure is perturbed due to envelope stress, RcsF remains in the periplasm, free to interact with the inner membrane protein IgaA (8, 10–12) and thereby to activate the Rcs system (13). When activated, the Rcs system regulates the expression of genes controlling many functions, including production of the exopolysaccharide colanic acid (14). A previous study suggested that the Rcs system transcriptionally regulates cell division proteins, further supporting a connection between Rcs activation and cell shape (15). Moreover, after digestion of the cell wall with lysozyme, Rcs-deficient mutants are unable to divide or recover cell shape (16).

Environmental changes can themselves have varied impacts on bacterial cell shape and size, features that are connected with behaviors such as motility, adhesion, and immune evasion (17), all of which are regulated by the Rcs pathway (6). The nutritional content of the environment also strongly affects *E. coli* and *Salmonella enterica* cell size, with faster-growing cells having larger volume (2, 18). In transitions between nutrient-poor and nutrient-rich environments, *E. coli* cells rapidly adjust their length and width within an hour (19, 20). By contrast, during steady-state exponential growth, bacteria robustly maintain their cell shape and size (21). This cell-size homeostasis results from an adder mechanism of growth (22, 23), in which cells on average add a fixed volume ∆*V* during each cell cycle (24). However, the molecular mechanisms by which cells modulate their cell size during nutrient changes and how they are coupled to growth rate remain largely unknown.

Bacterial cell size and shape are determined by the peptidoglycan cell wall, a macromolecule composed of glycan strands cross-linked by peptides (25). During cell elongation, the actin homolog MreB controls the spatial pattern of cell-wall synthesis (26, 27) and is essential for maintaining rod-like shape in many bacteria, such as *E. coli* (26). Depletion of MreB (28) or depolymerization of MreB by the small molecule A22 results in cell rounding and eventual lysis (29). Additionally, sub-inhibitory concentrations of A22 cause cell width to increase and cell length to decrease (30) without affecting cell-wall composition (31). During cell division, cell-wall synthesis localizes to a ring at mid-cell, initiated by the essential and highly conserved tubulin homolog FtsZ (32, 33). The concentration of FtsZ is negatively correlated with growth rate (34–36), and overexpression results in decreased cell length (37). Moreover, FtsZ overexpression can restore viability to cells depleted of MreB (28), suggesting that FtsZ levels can impact both cell-shape regulation and survival.

Here, we investigate the physiological consequences of Rcs activation in both the absence and presence of cell-envelope damage. We show that induction of RcsF^IM^, a mutant variant of RcsF that localizes to the inner membrane and constitutively activates the Rcs pathway in the absence of cell envelope stress (8, 38), reduces growth rate and cell length, increases FtsZ concentration, and reduces the distance between division rings in filamentous cells. Depletion of IgaA resulted in similar phenotypes, suggesting a general response to Rcs activation in the absence of envelope stresses. Nonetheless, cells treated with A22 maintained growth rate and FtsZ concentration, even though the Rcs system was activated. Thus, FtsZ levels and cell length are likely downstream of the growth-rate decreases caused by Rcs activation. Altogether, our results show that the nature of an activating stress can affect the phenotypic consequences of the Rcs pathway, similar to the differential response of the EnvZ/OmpR pathway to pH versus osmolality (39).

## RESULTS

### Induction of a constitutively active RcsF mutant reduces growth rate and cell length

A22 and mecillinam treatment each make *E. coli* rounder, increasing cell width and decreasing cell length (30), and activate the Rcs pathway (8). As we show in an accompanying study, increases in cell width due to A22 treatment are linked to Rcs activation (40). However, in such conditions, it is hard to assess whether Rcs activation itself affects cellular dimensions and growth rate. To decouple the cue from the response, we reasoned that we should control Rcs activation ectopically. To do so, we used an RcsF^IM^ mutant, which was previously shown to result in constitutive Rcs activation due to RcsF’s mislocalization to the inner membrane, where it can bind to IgaA and activate Rcs signaling (8). All experiments with RcsF^IM^ were conducted in an ∆*rcsF* background to avoid any competition with the expression of wild-type RcsF.

**Fig 1:**
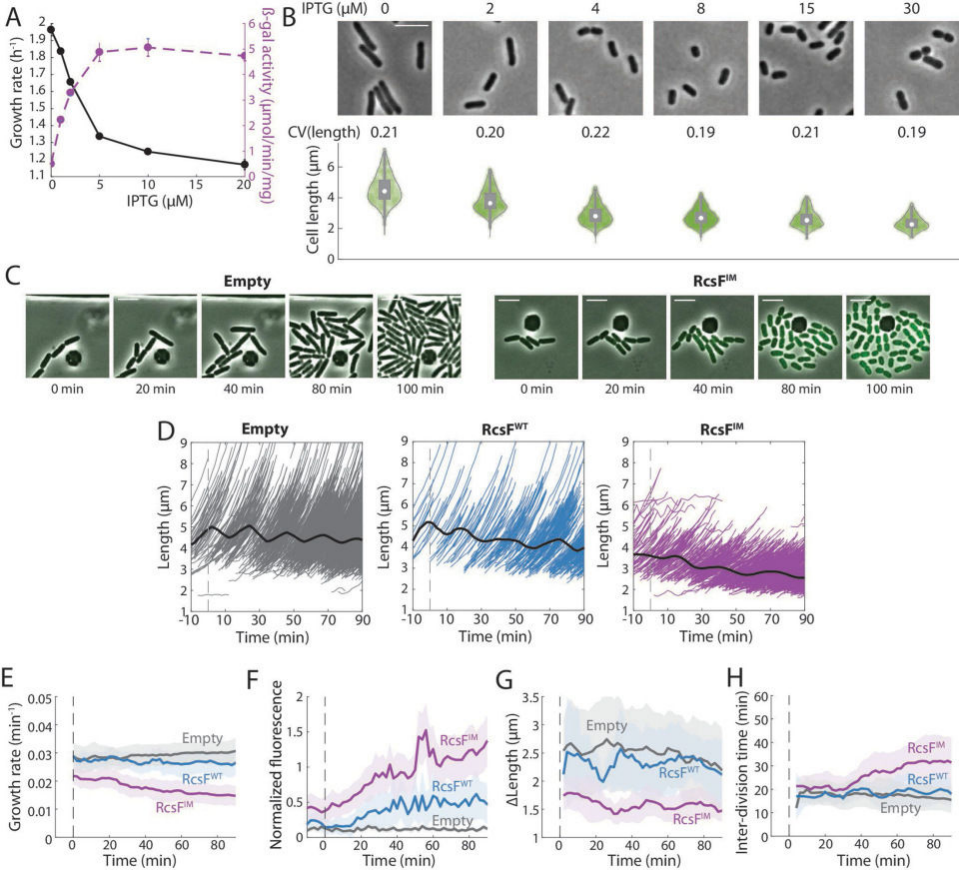
RscF activation in the absence of envelope stress alters cell-length control. (**A**) In batch culture, ∆*rcsF* cells expressing a constitutively active RcsF inner-membrane variant (RcsF^IM^) exhibited a decrease in steady-state growth rate dependent on the level of RcsF^IM^ expression and Rcs activation as measured by *β*-galactosidase activity. Error bars represent the standard deviation (SD) of *n* = 3 replicate experiments. (**B**) The length of RcsF^IM^-induced cells in batch culture decreased with increasing IPTG concentration. Top: representative cells. Bottom: violin plots of the distribution of cell lengths at each concentration, with *n* > 500 cells in each condition. *P* < 0.001 for all induced conditions compared to the no-induction control, two-tailed Student’s *t*-tests. White dots represent the median, gray bars represent the first and third quartiles of the data, and thick gray lines represent the 1.5 interquartile ranges. The coefficient of variation (CV) of cell length was similar across conditions. Scale bar: 5 µm. (**C**) After induction of RcsF^IM^ with 15 µM IPTG at *t* = 0 in a microfluidic chamber, RcsF^IM^ cells decreased in length relative to ∆*rcsF* cells with an empty vector and became physically separated. Scale bar: 5 µm. Cells were grown in LB. (**D**) RcsF^IM^ induction of the population shown in (**C**) at *t* = 0 resulted in a gradual reduction in mean cell length (solid black curve, right), while ∆*rcsF* cells (empty vector, left) maintained their mean length, and RcsF^WT^ induction resulted in a slight length decrease (middle). Gray, blue, and purple lines are trajectories of individual cells. *n* > 900 cell lineages for each strain. (**E**) Instantaneous growth rates (calculated as 1/*V dV*/*dt*, where *V* is cell volume) averaged across the populations in (**D**) show that RcsF^IM^ induction gradually reduced growth rate, while ∆*rcsF* cells maintained growth rate, and RcsF^WT^ induction resulted in an intermediate growth-rate reduction. (**F**) Mean msfGFP fluorescence from the *rprA* promoter averaged across the populations in (**D**) shows that Rcs was activated to a high and intermediate level in RcsF^IM^ and RcsF^WT^ cells, respectively, but not in ∆*rcsF* cells. (**G**) Cell length added over the course of each cell cycle (∆*L*) averaged across the populations in (**D**) stabilized at a lower value after induction of RcsF^IM^ compared to ∆*rcsF* or RcsF^WT^-induced cells. During 0–10 min, *P* < 0.001 between RcsF^IM^ and ∆*rcsF*, and *P* = 0.004 between RcsF^IM^ and RcsF^WT^. At the end of the experiment, *P* = 0.003 between RcsF^IM^ and ∆*rcsF*, and *P* < 0.001 between RcsF^IM^ and RcsF^WT^, two-tailed Student’s *t*-tests. (**H**) Division interval averaged across the populations in (**D**) shows that RcsF^IM^ induction gradually increased the time required for division, but not immediately after induction. During the first 10 min of induction, *P* = 0.68 between RcsF^IM^ and ∆*rcsF*, and *P* = 0.41 between RcsF^IM^ and RcsF^WT^; during the last 10 min of the experiment, *P* < 0.001 between RcsF^IM^ and ∆*rcsF* and between RcsF^IM^ and RcsF^WT^. *P* values based on two-tailed Student’s *t*-tests.

To quantify growth behavior and cellular dimensions during exponential growth, we expressed RcsF^IM^ from an isopropyl *β*-D-1-thiogalactopyranoside (IPTG)-inducible promoter on a low-copy plasmid in DH300 ∆*rcsF* cells (Table S1). We kept cells in steady state and depleted the Rcs proteins produced during the stationary phase (5) by continuously diluting RcsF^IM^ cells (Materials and Methods). At the same time, we added various concentrations of IPTG and monitored population growth rate, cell size, and Rcs activation levels via *β*-galactosidase activity of the promoter of *rprA*, a small RNA expressed when the Rcs pathway is activated (41). Growth rate decreased and *rprA* expression increased as a function of IPTG concentration, with *rprA* expression saturating at 5–10 µM IPTG as measured by *β*-galactosidase activity (Fig. 1A). The growth rate decrease was dependent on the presence of *rcsB* (Fig. S1A), indicating that it is caused by Rcs pathway activation. Concomitantly, cell length decreased (Fig. 1B). Maximum optical density in the stationary phase also decreased with increasing concentrations of IPTG (Fig. S1B). Thus, ectopic Rcs activation in the absence of envelope stress slows down growth, decreases cell size, and reduces total biomass production.

To determine the dynamics of RcsF activation on single cells, and to avoid confounding effects of cell shape changes on optical density measurements, we performed time-lapse imaging in a microfluidic flow cell (Materials and Methods) that enabled precise control of induction timing via switching to medium supplemented with IPTG. In this case, we utilized ∆*rcsF* strains with a plasmid expressing wild-type RcsF (RcsF^WT^) or RcsF^IM^ from an IPTG-inducible promoter (or an empty vector control), and a second plasmid carrying *msfGFP* under the *rprA* promoter to quantify Rcs activation (41). Cells were grown for 10 min in LB before supplementation of the medium with 15 µM IPTG to saturate Rcs activation by RcsF^IM^ (Fig. 1A). Upon RcsF^IM^ induction from the first plasmid, cell length (Fig. 1C and D) and single-cell instantaneous growth rate (measured as 1*/V dV*/*dt*, where *V* is cell volume, Fig. 1E) decreased steadily, while the empty-vector control strain remained largely unaffected (Fig. 1D and E). The Rcs pathway was activated in RcsF^IM^-induced cells (Fig. 1F). RcsF^WT^-induced cells exhibited a small reduction in cell length (Fig. 1D) and growth rate (Fig. 1E), consistent with the observation that RcsF^WT^-induced cells exhibited msfGFP levels intermediate between empty vector and RcsF^IM^ cells (Fig. 1F) and with previous findings that RcsF overproduction induces Rcs activation (14).

We tracked cell lineages in our time-lapse data to determine whether the volume added during the cell cycle was affected by Rcs activation. Upon IPTG addition, the length added over the course of a cell cycle ∆*L* (a proxy for added volume) in RcsF^IM^ cells equilibrated to a value 30%–40% lower than that of empty-vector or RcsF^WT^ cells (Fig. 1G), consistent with observations of lower mean cell length (Fig. 1D) and lower growth rate (Fig. 1E). Interestingly, cell-cycle duration increased by 30%–40% over the 90 min of induction (Fig. 1H). Taken together, these data indicated that Rcs activation alters growth rate, cell size, and cell-cycle timing, which motivated us to further investigate the link between Rcs activation and cell cycle.

#### Cell shape and growth rate changes are not due to colanic acid production

**Fig 2:**
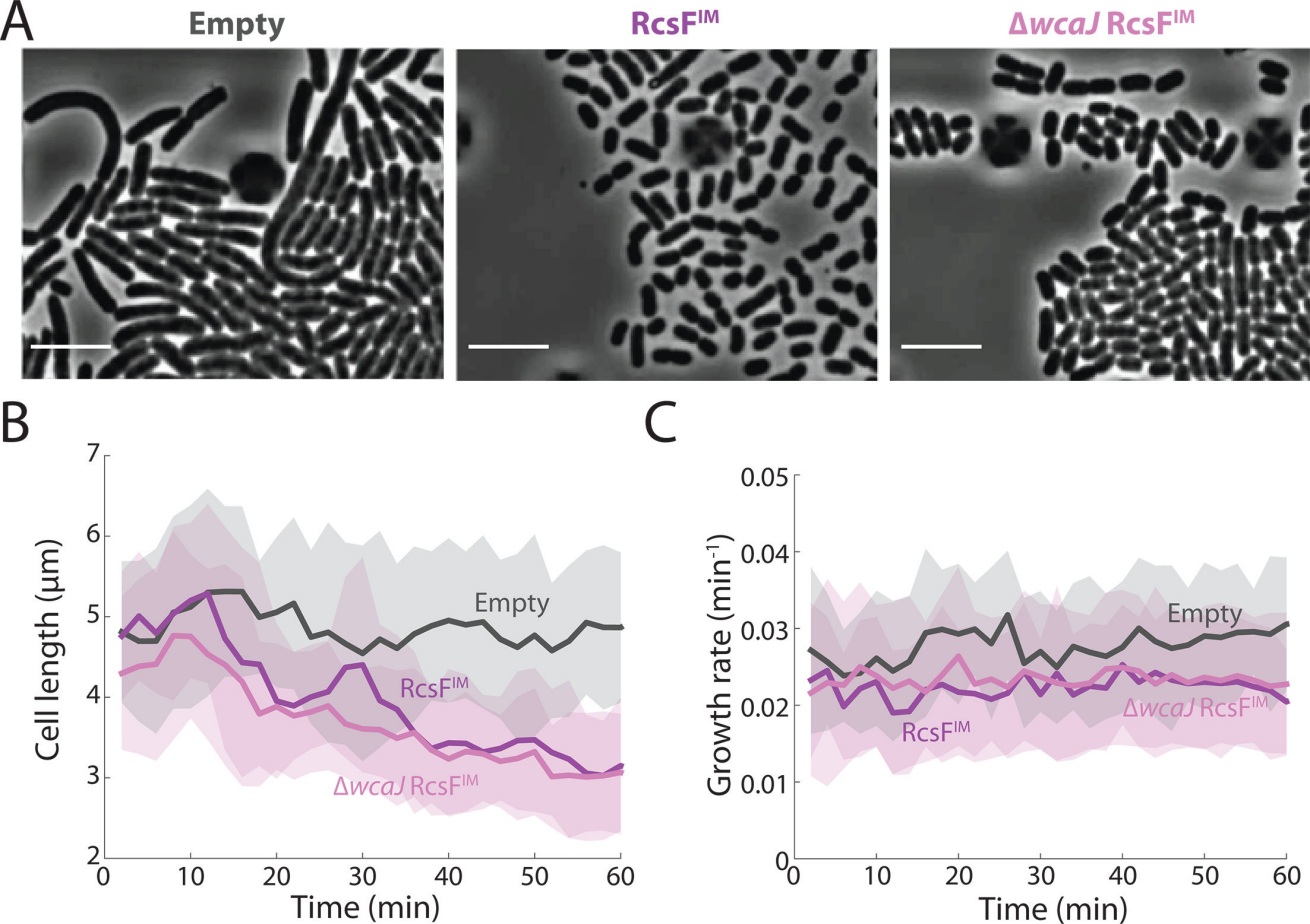
Colanic acid production is not responsible for the changes in cell length and growth rate observed upon RcsF activation. (**A**) Time-lapse imaging of a mutant lacking *wcaJ*, which encodes the most upstream component of colanic-acid synthesis, after induction of RcsF^IM^ with 15 µM IPTG in LB media at *t* = 0. Unlike wild-type cells (middle), ∆*wcaJ* cells (right) remained tightly packed, similar to ∆*rcsF* cells with an empty vector (left), which do not activate the Rcs system. Scale bar: 10 µm. (**B and C**) Cell length (**B**) and growth rate (**C**) trajectories for ∆*wcaJ* cells were similar to those of RcsF^IM^-induced (15 µM IPTG) wild-type cells. *n* > 690 cell lineages for each strain. Growth rate was calculated as in Fig. 1E.

Production of the exopolysaccharide colanic acid is regulated by the Rcs pathway (42). In our time-lapse imaging experiments, RcsF^IM^-induced cells became highly separated over time (Fig. 1C and 2A), which we inferred was due to the production of colanic acid. We hypothesized that colanic acid production could entail a substantial metabolic cost and result in the reductions in cell length (Fig. 1D) and growth rate (Fig. 1E) observed upon Rcs activation. To test this hypothesis, we deleted *wcaJ*, the most upstream gene in the colanic acid synthesis pathway that encodes the initial lipid carrier. Knocking out colanic acid biosynthesis through disruption early in the pathway avoids the buildup of intermediates, which can alter cell shape due to titrating precursor flux away from cell-wall synthesis (43). Using a microfluidic flow cell, we performed time-lapse imaging of RcsF^IM^-induced ∆*wcaJ* cells. ∆*wcaJ* cells remained closely packed (Fig. 2A), supporting the idea that the cell separation is due to colanic acid. ∆*wcaJ* cells showed a similar decrease in mean cell length (Fig. 2B) and growth rate (Fig. 2C) as colanic acid-producing cells (Fig. 1D and E), indicating that colanic acid production is not the cause of the cell shape and growth rate changes upon RcsF^IM^ induction.

#### Constitutive Rcs activation increases FtsZ intracellular concentration and FtsZ localization to division sites

**Fig 3:**
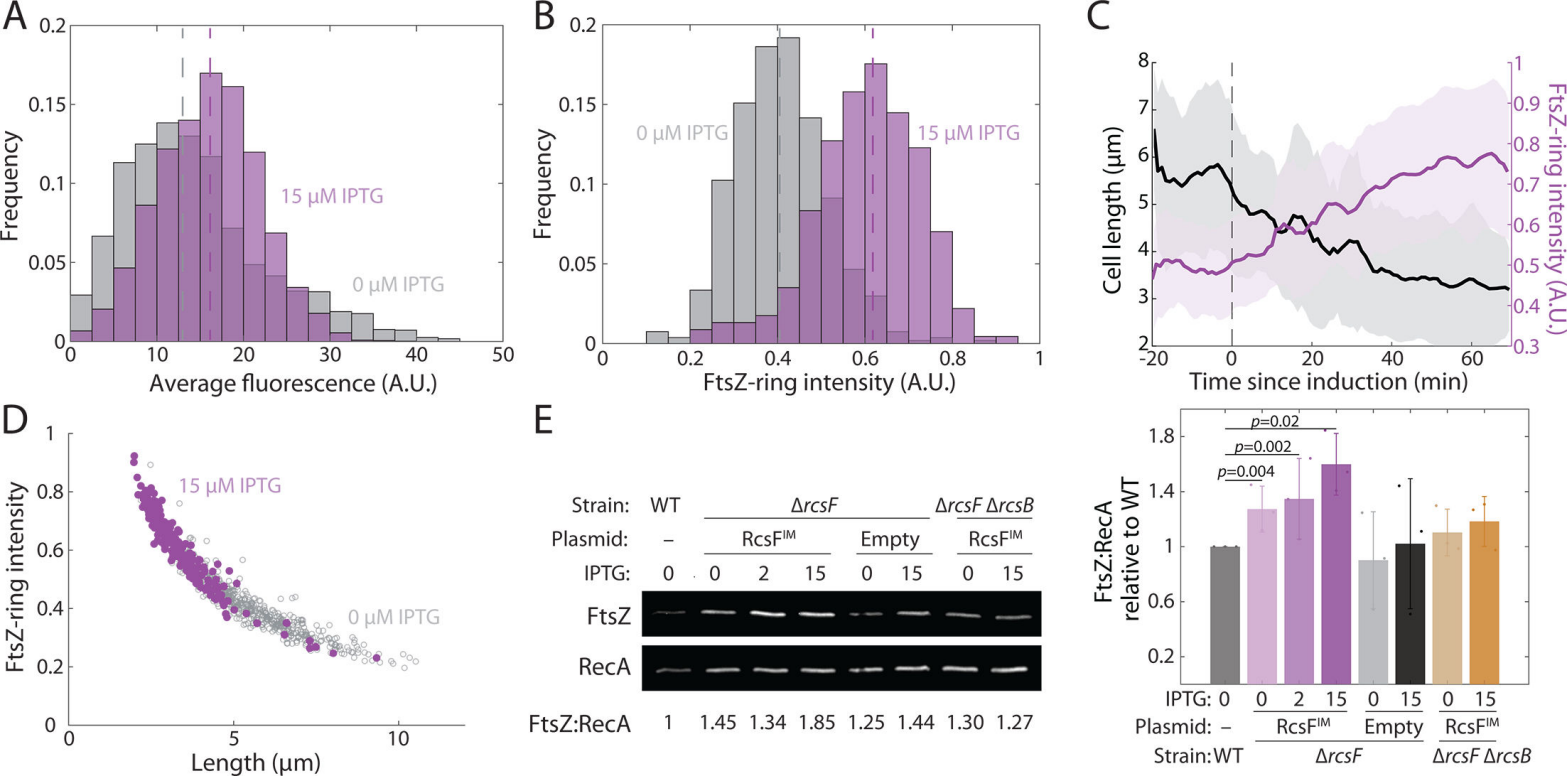
RcsF^IM^ induction results in increased FtsZ cellular and mid-cell concentration. (**A and B**) RcsF^IM^ induction with 15 µM IPTG for 60 min increases FtsZ-msfGFP fluorescence intensity (total fluorescence normalized to cell volume) (**A**) and FtsZ-ring concentration (**B**) relative to uninduced cells. Dashed lines represent median values. *P* < 0.001 in both panels, two-tailed Student’s *t*-tests. *n* > 200 cells for each induction level. Cells were grown in LB and imaged on agarose pads with M9 glucose to minimize background fluorescence. (**C**) FtsZ-ring intensity (purple) gradually increased as cell length (black) decreased due to RcsF^IM^ induction with 15 µM IPTG at *t* = 0. *n* = 12 cells. Cells were grown and imaged as in panel A. (**D**) For both uninduced and induced RcsF^IM^ cells, cell length followed an inverse relationship with FtsZ-ring intensity that overlapped between the two induction conditions. *n* > 200 cells for each condition. Cells were grown and imaged as in panel **A**. (**E**) Western blotting demonstrated that the ratio of FtsZ to RecA levels increased in cells in which RcsF^IM^ was induced, but not in cells with an empty vector or cells lacking *rcsB*. Left: a representative Western blotting gel (replicates are shown in Fig. S1C). Right: bars are mean ± 1 SD of *n* = 3 replicates (shown as individual dots). *P* values are from one-tailed Student’s *t*-tests.

A previous study showed that RcsB, the primary activator of Rcs-regulated genes, can bind upstream of one of the many *ftsZ* promoters (15) and activate the *ftsAZ* operon, suggesting that the Rcs system regulates production of the division machinery. To assess this scenario, we quantified FtsZ levels upon RcsF^IM^ induction and related expression to changes in growth rate and cell length. To quantify total cellular FtsZ concentration as well as the concentration specifically within the FtsZ-ring, we introduced an FtsZ-msfGFP sandwich fusion into the chromosome at the native *ftsZ* locus in *E. coli* DH300 ∆*rcsF* cells carrying each RcsF plasmid variant. Cells expressing the *ftsZ-msfGFP* fusion as the sole copy of *ftsZ* are viable (44) and have similar growth rates as cells with native *ftsZ* (45), although mean cell length increases due to a reduction in FtsZ’s GTPase activity (46). We imaged cells on agarose pads with M9+0.04% glucose (to alleviate the high autofluorescence of LB) after supplementation with 0 or 15 µM IPTG. msfGFP intensity (total fluorescence normalized to cell volume) was higher in RcsF^IM^-induced compared to uninduced cells (Fig. 3A; median fluorescence: 13.0 for 0 µM IPTG and 16.2 for 15 µM IPTG; mean fluorescence: 14.3 for 0 µM IPTG and 16.6 for 15 µM IPTG), indicating that Rcs activation increased FtsZ levels, and RcsF^IM^-induced cells exhibited a ~50% increase in FtsZ-ring intensity relative to uninduced cells (Fig. 3B). During time-lapse imaging in a microfluidic flow cell, FtsZ-ring intensity increased over time in RcsF^IM^-induced cells, concurrent with a decrease in mean cell length (Fig. 3C). Across a population of single cells, cell length and FtsZ-ring intensity followed a tightly constrained inverse relationship with both 0 and 15 µM IPTG (Fig. 3D). Thus, we inferred that decreases in cell length were coupled to local increases of FtsZ levels within the FtsZ-ring.

To verify the increase in FtsZ concentration, we directly measured FtsZ protein levels with antibodies. We analyzed wild-type, ∆*rcsF* strains with the empty and RcsF^IM^ plasmids, and a ∆*rcsF* ∆*rcsB* strain with the RcsF^IM^ plasmid (to confirm dependence on the Rcs system). Strains were diluted and passaged multiple times to ensure exponential growth and then treated with 0, 2, or 15 µM IPTG for 90 min. From Western blotting, we calculated the ratio of FtsZ to RecA levels (as a control) and normalized to that of wild type. Basal expression of RcsF^IM^ slightly increased the FtsZ/RecA ratio, and addition of 2 or 15 µM IPTG led to a further increase of ~50% (Fig. 3E; Fig. S1C), approximately consistent with our fluorescence measurements (Fig. 3A). This increase required the activation of Rcs-controlled genes by RcsB (Fig. 3E). Thus, Rcs activation leads to an increase in FtsZ levels.

#### Rcs induction decreases the separation between FtsZ rings in filamentous cells

**Fig 4:**
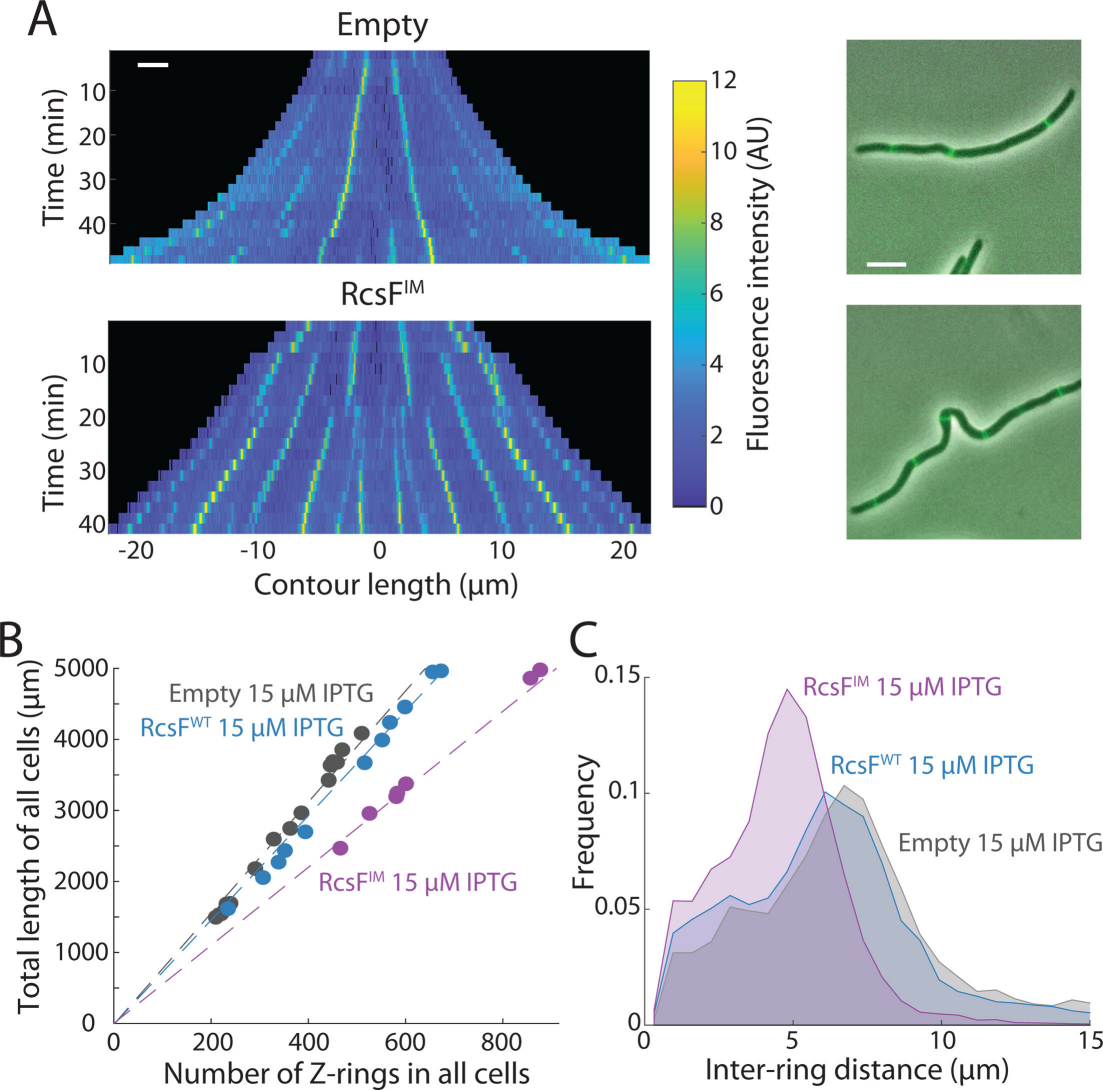
RcsF^IM^ induction decreases the distance between FtsZ rings in filamentous cells. (**A**) After cephalexin treatment to inhibit division, kymographs (left) and overlays of FtsZ-msfGFP fluorescence and phase-contrast images at *t* = 40 min (right) after the start of induction with 15 µM IPTG show that the distance between FtsZ rings was shorter in RcsF^IM^-induced cells (bottom) compared to empty-vector ∆*rcsF* cells (top). Scale bars: 5 µm. Cells were grown and imaged as in Fig. 3. (**B**) The slope of the summed length of all cells in a field of view versus the number of Z-rings within those cells was lower in RcsF^IM^-induced cells, indicating that the number of rings per unit length was higher in RcsF^IM^ cells. Each circle represents a field of view. *n* = 21–255 cells for each field of view, *n* > 6 fields of view for each strain. Cells were grown and imaged as in Fig. 3. (**C**) The distance between FtsZ rings was significantly shorter in RcsF^IM^-induced cells compared to RcsF^WT^-induced cells and empty-vector ∆*rcsF* cells, *P* < 0.001 in both cases, two-tailed Student’s *t*-tests. *n* > 1,500 cells for each strain. Cells were grown and imaged as in Fig. 3.

Normally, dividing cells harbor a single FtsZ ring positioned at midcell, regardless of cell length (34). To determine whether FtsZ localization dynamics and local variations in FtsZ concentration were affected by RcsF^IM^ induction and connected with reductions in cell length, we treated cells with cephalexin, a beta-lactam that inhibits the division-specific transpeptidase FtsI (47). These filamentous cells had multiple FtsZ rings that were easily identified based on the peaks in peripheral fluorescence (Materials and Methods) (45). After growth on LB agarose pads with 15 µM IPTG and cephalexin, kymographs indicated that RcsF^IM^-induced cells had more closely spaced FtsZ rings than empty-vector cells (Fig. 4A). Indeed, RcsF^IM^-induced cells exhibited an increased number of rings per unit length (Fig. 4B) and decreased distance between FtsZ rings (Fig. 4C) compared with empty-vector or RcsF^WT^-induced cells. Thus, RcsF^IM^ induction increases the number of FtsZ rings per unit length in cephalexin-treated cells, thereby defining shorter cellular units even in the absence of cytokinesis.

#### IgaA depletion mimics RcsF^IM^ induction

**Fig 5:**
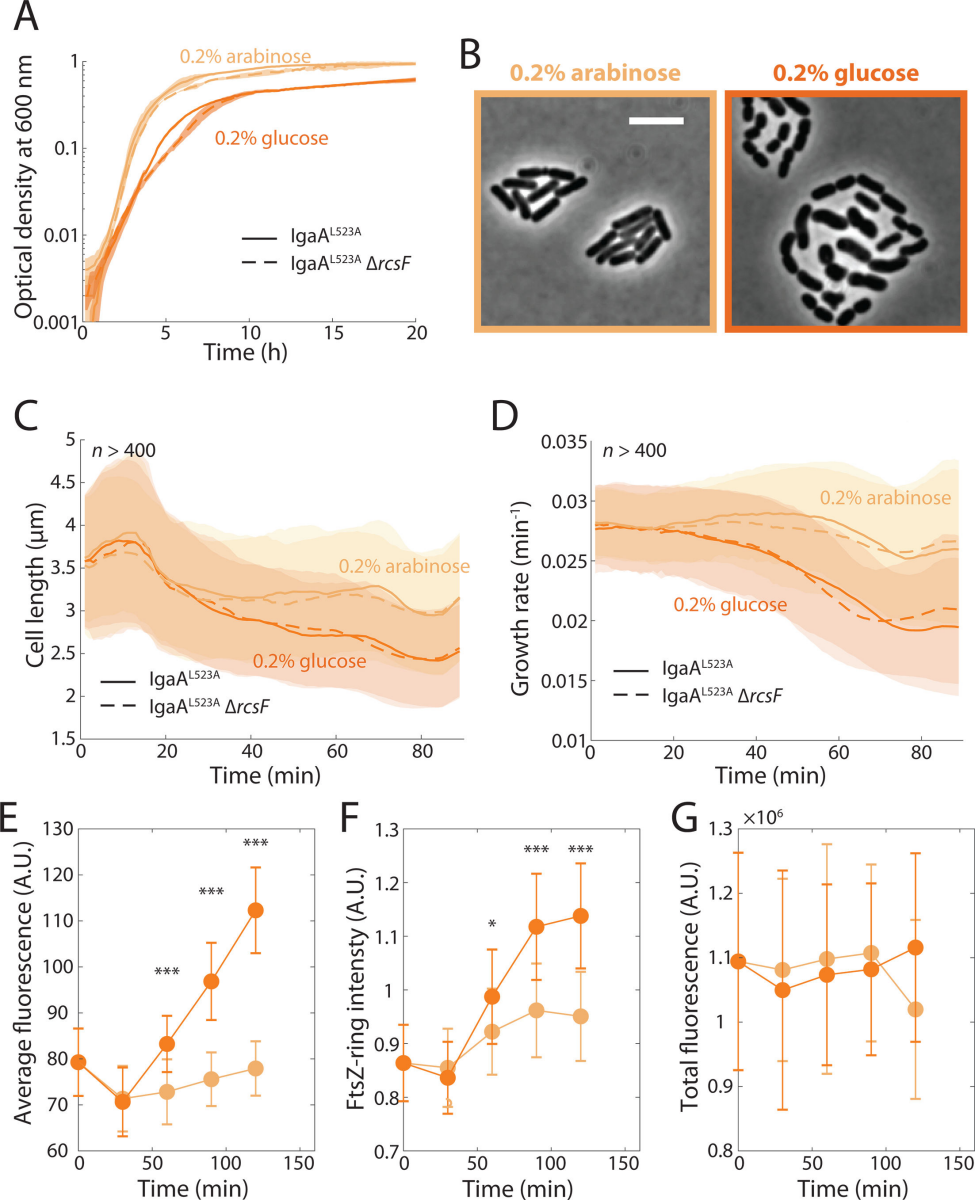
IgaA^L523A^ depletion results in similar phenotypes to RcsF^IM^ induction. (**A**) Using a strain with a plasmid expressing the *igaA^L523A^* allele from an arabinose-inducible promoter with the native *igaA* deleted (KC1183, Table S1), depleting IgaA^L523A^ with glucose resulted in a reduction in growth. For final OD_600 nm_, *P* < 0.001 between glucose and arabinose conditions in both backgrounds, two-tailed Student’s *t*-tests. Shaded areas represent 1 SD across *n* = 12 replicates. Cells were grown in LB with additional glucose or arabinose. (**B**) IgaA^L523A^ depletion (glucose) resulted in shorter cells that became physically separated, indicating that the Rcs system is activated, unlike cells grown on arabinose. Images were taken 60 min after cells were placed on agarose pads. Scale bar: 5 µm. (**C and D**) IgaA^L523A^ depletion (glucose) caused cell length (**C**) and growth rate (**D**) to decrease, independent of RcsF, which lies upstream of IgaA in the Rcs signaling cascade. Growth rate was calculated as Fig. 1E. Comparing data with (KC1183) and without *rcsF* (KC1185, Table S1) between 60 and 80 min, length (glucose, *P* = 0.34; arabinose, *P* = 0.12) and growth rate (glucose, *P* = 0.61; arabinose, *P* = 0.31) were not significantly different, while *P* < 0.001 for all comparisons between glucose and arabinose. *P-*values are from two-sided Student’s *t*-tests with *n* > 500 cells in each condition. Data points are mean ± 1 SD. (**E–G**) In strains expressing an FtsZ-msfGFP sandwich fusion, average fluorescence normalized to cell volume (**E**) and FtsZ-ring intensity (F, Materials and Methods) increased upon IgaA depletion. Total fluorescence (**G**) remained similar in arabinose and glucose. Data points are mean ± 1 SD, with *n* > 50 cells in each condition. *: *P* < 0.05, ***: *P* < 0.001, two-tailed Student’s *t*-tests. For (**E–G**), cells were grown and imaged as in Fig. 3.

To determine whether the changes in growth rate (Fig. 1E), cell length (Fig. 1D), and FtsZ concentration (Fig. 3) were general features of Rcs activation as opposed to an artifact of RcsF^IM^ expression, we induced the Rcs system in a distinct manner, this time using the essential protein IgaA, which is an inner membrane-localized inhibitor of the Rcs system (10, 12). We first tested a strain expressing *igaA* from an arabinose-inducible promoter, with the native *igaA* deleted. When arabinose was removed, we observed a mild increase in Rcs activity, but cell growth was largely unaffected over several hours (Fig. S2). To obtain stronger effects of depletion, we replaced the wild-type *igaA* allele with *igaA^L523A^*. This allele, akin to the previously employed *igaA^L643P^* allele (8, 48), exhibits lower levels of Rcs repression (the L643P mutant is somewhat less stable and hence its depletion overcomes the basal promoter expression), and the effects of depletion are observed more readily than depletion of wild-type IgaA. To determine whether IgaA^L523A^ depletion and the resulting activation of the Rcs system affect FtsZ concentration, we transduced the FtsZ-msfGFP sandwich fusion into the chromosome of the *igaA^L523A^*-inducible strain. We monitored growth via absorbance in LB supplemented with either 0.2% arabinose, to maintain normal growth, or 0.2% glucose, to deplete IgaA^L523A^. With arabinose, the IgaA^L523A^ strain grew similarly to wild-type *E. coli*, while depletion with glucose substantially reduced growth rate (1.76 ± 0.17 h^−1^ in arabinose versus 1.15 ± 0.20 h^−1^ in glucose; *P* < 0.001, two-tailed Student’s *t*-test) (Fig. 5A), similar to the decrease in growth rate upon RcsF^IM^ induction (Fig. 1A). As expected, deletion of *rcsF* did not have an effect on growth (Fig. 5A), since IgaA functions downstream of RcsF.

We next examined the IgaA^L523A^ strain during depletion using single-cell microscopy. On agarose pads with 0.2% glucose, cells physically separated during growth and division, signifying colanic acid production due to Rcs activation (Fig. 5B). Mean cell length (Fig. 5C) and instantaneous growth rate (Fig. 5D) decreased in a manner similar to RcsF^IM^ induction (Fig. 1C and D). FtsZ-msfGFP fluorescence intensity (Fig. 5E) and FtsZ-ring intensity (Fig. 5F) increased coincident with the decrease in length (Fig. 5C), such that mean total fluorescence per cell remained approximately constant (Fig. 5G). In sum, the phenotypes that emerged during RcsF^IM^ induction were closely mimicked by IgaA^L523A^ depletion, suggesting that they are general properties of Rcs activation in the absence of envelope stress.

#### A22 activates RcsF without affecting growth rate or FtsZ concentration

**Fig 6:**
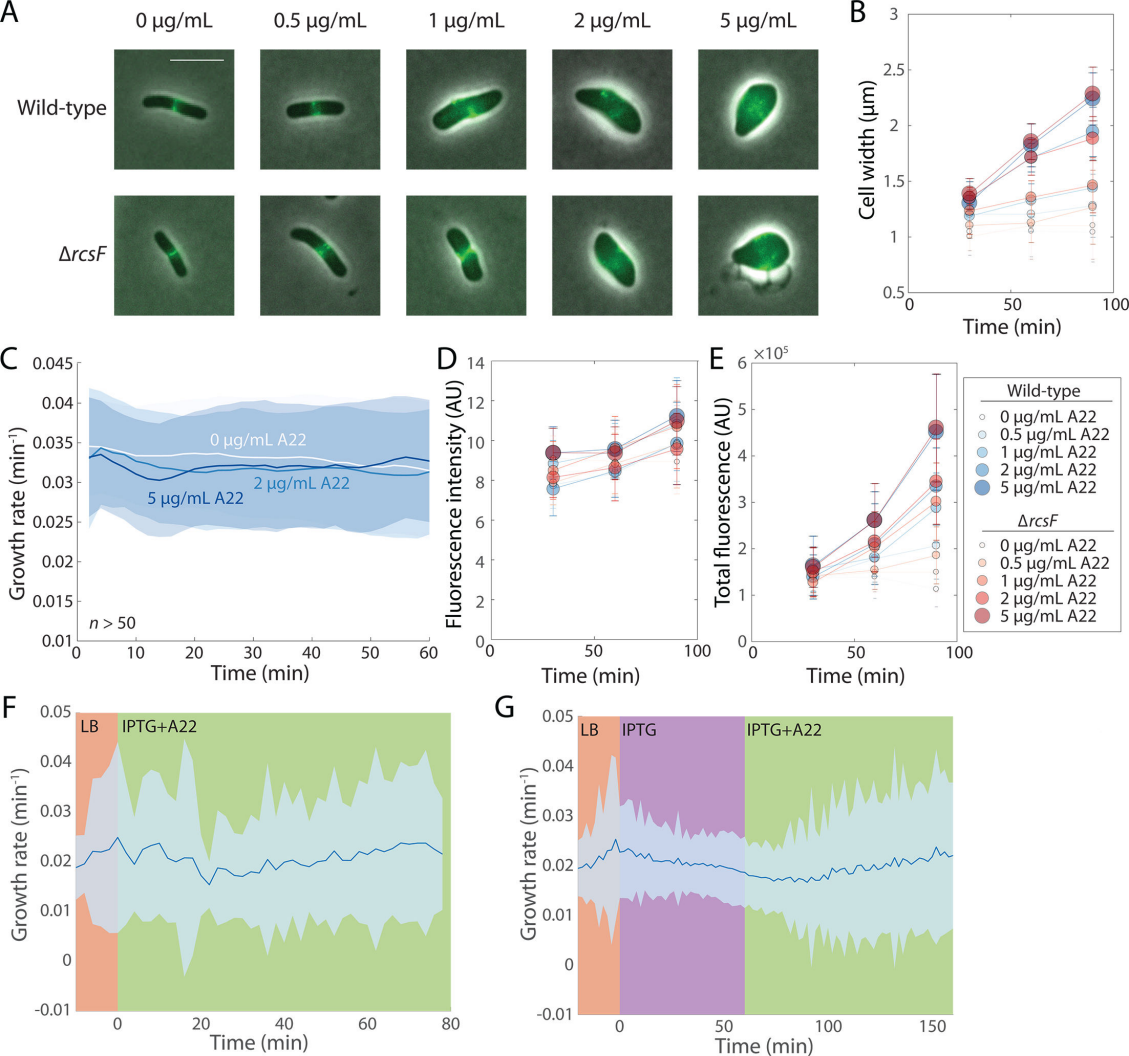
A22 treatment does not result in changes to growth rate or FtsZ concentration. (**A**) A22 treatment caused the width of both wild-type and ∆*rcsF* cells to increase. FtsZ-msfGFP fluorescence is overlaid on phase-contrast images. Images were taken 90 min after the start of A22 treatment. Scale bar: 5 µm. Cells were grown and imaged as in Fig. 3. (**B**) Cell width increased in an A22 dose-dependent manner, with similar increases in wild-type and ∆*rcsF* cells. A22 was added at *t* = 0. *n* > 50 cells per condition. (**C**) Log-phase wild-type cells grown in LB were placed onto LB-agarose pads containing different concentrations of A22, and growth was monitored by time-lapse imaging. Growth rate was calculated as in Fig. 1E. Throughout the experiment, growth rate was unaffected by A22 treatment. (**D**) A22 was added to log-phase wild-type or ∆*rcsF* cells in liquid LB at *t* = 0. Every 30 min, aliquots of cells were imaged on agarose pads to monitor changes to cell morphology and FtsZ intracellular intensity (total fluorescence normalized by cell volume). FtsZ-msfGFP fluorescence intensity in wild-type and ∆*rcsF* cells increased only slightly or not at all and was similar across all A22 concentrations. (**E**) Total FtsZ-msfGFP fluorescence in wild-type and ∆*rcsF* cells increased over time, reflecting the increase in cell width and volume (**B**) and maintenance of FtsZ-msfGFP intensity (**D**). (**F**) When simultaneously treated with 15 µM IPTG and 5 µg/mL A22, RcsF^IM^-induced cells maintained their growth rate (*P* = 0.08) in LB. (**G**) When RcsF^IM^ cells were pre-induced with 15 µM IPTG for 1 h, growth rate decreased (*P* < 0.001). Nonetheless, when 5 µg/mL A22 was added, growth rate gradually recovered pre-induction levels (*P* = 0.56). Data in (**B, D, E, F, G**) are mean ± 1 standard deviation (SD). *n* > 50 cells were analyzed for each condition. *P* values in (**F and G**) are from two-tailed Student’s *t*-tests, comparing the growth rates in the last 10 min of each condition.

To investigate the role of the Rcs system in cell growth and physiology during activation of cell envelope stress, we used time-lapse microscopy to analyze the response of wild-type cells to a range of A22 concentrations (Fig. 6A). As expected and consistent with the effect of A22 on cell elongation, mean cell width increased upon exposure to A22 in a dose-dependent manner (Fig. 6B) (31). However, despite rapid activation of the Rcs system by A22 (40) to similar levels as RcsF^IM^ induction (Fig. S3), growth rate was essentially maintained for the first hour (Fig. 6C), consistent with previous studies showing that A22 does not affect growth rate (49, 50).

This growth rate maintenance is markedly distinct from other Rcs-activating perturbations, such as RcsF^IM^ induction (Fig. 1E) or IgaA^L523A^ depletion (Fig. 5D). Moreover, there was little increase in the volume-normalized FtsZ-msfGFP intensity (Fig. 6D), as both total fluorescence and cell width increased together, approximately threefold for the former (Fig. 6E) and ~75% for the latter (Fig. 6B) at the highest A22 concentration. These phenotypes are also distinct from the increase in FtsZ concentration (Fig. 3A, E and 5E) observed for Rcs activation in the absence of envelope stresses. Thus, activation of Rcs by A22 does not result in the growth inhibition or upregulation of FtsZ that occurred when Rcs was activated through RcsF^IM^ induction or IgaA depletion.

To examine crosstalk between multiple Rcs-activating cues, we simultaneously treated cells with 5 µg/mL A22 and induced RcsF^IM^ expression with 15 µM IPTG. Unlike in the absence of A22, RcsF^IM^-induced cells maintained their growth rate (Fig. 6F), suggesting that A22 prevents the growth rate decrease that would otherwise result from Rcs activation by RcsF^IM^. To confirm that this result was not due to A22 preventing RcsF^IM^ induction, we pre-induced cells with 15 µM IPTG for 1 h before adding 5 µg/mL A22. During pre-induction, growth rate decreased as expected (Fig. 6G). Nonetheless, once A22 was added, growth rate recovered back to pre-induction levels (Fig. 6G). Thus, A22 treatment is dominant over Rcs activation by RcsF^IM^ induction.

In line with the maintenance of FtsZ concentration (Fig. 6D) despite activation of the Rcs system under A22 treatment (40), the trajectories of cell width (Fig. 6B) and growth rate (Fig. 6C) were quantitatively similar in A22-treated wild-type and ∆*rcsF* cells, indicating that the morphological effects of A22 treatment (cell widening) are independent of the Rcs pathway. Consistent with this finding, wild-type and ∆*rcsF* cells exhibited similar shape trajectories during recovery from A22 (Fig. S4). Given that FtsZ concentration varies with nutrient-determined growth rate (34), these data suggest that the dynamics of cellular dimensions and FtsZ are downstream of the growth-rate effects of Rcs activation and, more generally, that the downstream responses of a signaling pathway can depend on the activating stress.

## DISCUSSION

In this study, we showed that activation of the Rcs system in the absence of envelope stress adjusts the added length (Δ*L*) per cycle and decouples elongation and cell-cycling timing, leading to shorter cells (Fig. 1C through F). This phenotype is achieved by up-regulation of FtsZ levels and FtsZ ring formation (Fig. 3A, B and E), which likely are consequences of the slowdown in growth due to Rcs activation (34). The up-regulation in FtsZ protein levels may be due in part to the effect of Rcs on *ftsAZ* transcription (15, 51); how FtsZ increases its mid-cell localization remains to be elucidated. Overall, our data highlight the strong coupling between growth rate and FtsZ levels, the latter of which determines cell length in a highly stereotyped manner (Fig. 3D).

The growth rate decrease and upregulation of FtsZ in RcsF^IM^-induced cells (Fig. 1D and E) is a direct result of Rcs activation, as IgaA depletion had similar effects (Fig. 5D and E). Thus, these phenotypes are likely to be general consequences of Rcs activation in the absence of envelope stresses. However, A22 treatment did not affect growth rate (Fig. 6C) or FtsZ concentration (Fig. 6D) despite activation of the Rcs pathway (40). Unlike the specificity of activation by RcsF^IM^ induction and IgaA depletion, A22 also induces other envelope-stress responses such as the Cpx system (52), which may buffer or counteract the growth defects driven by Rcs alone and thus contribute to the distinct phenotypic outcome. Moreover, A22 treatment resulted in cell-length decreases similar to RcsF^IM^ induction even in the absence of RcsF (Fig. S5), indicating that there are Rcs-independent mechanisms for coupling the cell cycle to cellular dimensions (18, 53), and/or there may be additional signals that buffer or alter the Rcs response during cell elongation stress. One possible implication of our observations is that the Rcs pathway serves as a sensor of nutritional status. During nutrient shifts, changes to envelope synthesis and turgor could transiently alter periplasmic thickness, modulating Rcs activity and FtsZ levels. In such a scenario, Rcs would integrate mechanical cues arising from metabolic changes to help regulate size changes across nutrient environments, an intriguing avenue for future work. Regardless, these findings indicate that the results of activating a stress response can be highly dependent on context.

Our results also provide a plausible explanation for why *igaA* deletions are non-viable in Enterobacteriaceae family members, unless Rcs is also knocked out or attenuated through decreased expression of RcsC or RcsD (8, 48, 51). As we have shown, the resulting constant activation of the Rcs pathway leads to cells becoming very small, likely tuning Δ*L* below a level that supports growth. The terminal phenotypes due to RcsF^IM^ induction or IgaA depletion are distinct from the effects of depletion of many other essential proteins in *E. coli* (54). Interestingly, LPS-related stress that induces Rcs independently of cell size cues (40) causes transient Rcs activation that fixes the damage (55, 56).

Our inner membrane-targeted RcsF mutant constitutively activates the Rcs phosphorelay by bypassing the normal outer-membrane assembly steps and initiating downstream signaling. In a companion study, we found that inhibition of MreB by A22, which disrupts the pattern of cell-wall insertion and alters cell shape, and other mechanical or genetic perturbations that change cell width lead to Rcs activation (40). The mean cell width of MreB mutants or of wild-type cells treated with A22 was correlated with the degree of Rcs pathway activity, and cryoelectron tomograms revealed a thinner periplasm in a wider MreB mutant (40). We thus speculate that the increase in cell width due to A22 treatment also decreases periplasmic thickness. Such a change could result from uneven peptidoglycan architecture, which perturbs the normal membrane contacts that keep RcsF inactive, or through size-mediated alteration to the balance of forces across the envelope.

Our results also provide insights into the potential benefits of Rcs pathway activation during envelope stress. By increasing FtsZ levels and thereby sustaining FtsZ ring formation even as elongation slows, cells can counteract the filamentation that often accompanies envelope damage and can lead to catastrophic breakage (57). Moreover, more frequent septation partitions any localized envelope damage into smaller progeny, acting as a bet-hedging strategy that increases the chance that at least some offspring survive severe stress. Finally, in complex settings such as biofilms or host tissues, smaller, more numerous cells may better negotiate tight spaces and maintain high surface area-to-volume ratios for nutrient uptake and waste removal, enhancing overall population resilience.

The relationship between the Rcs pathway and cell division has long been the subject of speculation, with overexpression of RcsB and RcsF previously shown to suppress the division defect of an *ftsZ84* mutant (58, 59). Analysis of the *ftsZ* promoter sequence identified an RcsB binding site that enables transcription of *ftsZ* upon Rcs induction (15). Our findings present a more nuanced perspective on Rcs-mediated regulation of the division machinery, in which increases in FtsZ concentration occur only for conditions in which growth rate decreases, such as RcsF^IM^ induction (Fig. 1D) or IgaA depletion (Fig. 5D) but not A22 treatment (Fig. 6C). Taken together, our findings reinforce previous studies linking the elongation and division machineries (45, 60, 61) and highlight the importance of growth rate in determining cell size.

Understanding how the Rcs system enacts physiological changes in bacterial cells has important implications for the general response of bacteria to stresses. It remains unclear why deletion of *rcsF* sensitizes *E. coli* to A22 treatment in the context of growth as a colony (7). Our observations that log-phase ∆*rcsF* cells exhibit the same morphological and growth response to A22 treatment (Fig. 6A through C) and subsequent recovery trajectory (Fig. S4) as wild-type cells suggest that the sensitivity may arise in lag or stationary phase, manifesting as growth delays or lower yield. Regardless, one significant takeaway from our study is that cell shape and transcriptional changes can result from a global change in growth rate rather than directly from stress-response pathway activation. That cells maintain growth rate during A22 treatment in the face of Rcs activation (even by RcsF^IM^ induction, Fig. 6F and G) suggests that other responses induced by A22 interfere with some of the downstream Rcs signaling. It will be interesting to probe the extent to which activation of stress response pathways generally leads to changes in growth rate; our findings predict that activation of growth-inhibiting pathways will also cause *ftsZ* upregulation. Moreover, changes in growth rate could lead to cross-protection in the presence of other stresses (62); for example, growth in minimal medium alleviates the essentiality of MreB (63). Ultimately, our strategy of decoupling Rcs activation from the activating stresses should be a powerful strategy for connecting response pathways to their direct effects on physiological phenotypes.

## MATERIALS AND METHODS

### Strains, plasmids, and media

All strains and plasmids are in Table S1. *E. coli* MG1655 and its derivatives were used in all experiments.

To construct RcsF^IM^, the serine at position 17 was replaced with aspartic acid (S17D) through site-directed mutagenesis with pSC202 as a template (8, 55).

For plasmid replicons and promoters, the appropriate *rcsF* allele was cloned between the EcoRI and HindIII sites of the low-copy pNG162 vector (pSC101 origin with the *trc* promoter) (64). All single-gene deletions were introduced into wild-type *E. coli* via P1 transduction.

We used P1 transduction to introduce *ftsZ-msfGFP* into the chromosome at the native *ftsZ* locus of DH300 ∆*rcsF* cells, and introduced the pNG162-empty, pNG162-WT, and pNG162-IM plasmids harboring the RcsF variants, along with the pTrcHis2A plasmid, which carries *lacI*^q^ from a high-copy plasmid to shut down basal expression from the pNG162 plasmid (8).

Unless otherwise specified, cells were cultivated in LB Lennox (10 g/L tryptone, 5 g/L yeast extract, 5 g/L NaCl). For imaging of FtsZ fusions, M9 (65) media were used. Antibiotics were added to the culture when needed to maintain plasmids: 50 µg/mL for spectinomycin, 100 µg/mL for ampicillin, and 35 µg/mL for chloramphenicol.

### Growth-rate measurements for batch cultures

For Fig. 1; Fig. S1, overnight cultures were grown at 37°C in LB (Lennox formulation) with appropriate antibiotics. Cells were diluted to OD_578 nm_ = 0.001 and grown for ~3 h until OD ~0.1. Cells were then diluted to OD_578 nm_ = 0.025 in fresh LB with 0 to 15 µM IPTG. To enable growth-rate quantification, OD_578 nm_ was measured throughout the experiment, and once OD_578 nm_ = 0.3 was reached, cells were diluted to OD_578 nm_ = 0.025. This passaging cycle was maintained for 6 to 9 h. Growth rate was calculated from OD_578 nm_ measurements using ≥3 measurements from the range with OD_578 nm_ <0.4 by fitting to an exponential.

For the measurements in Fig. 5A, overnight cultures in LB with 100 µg/mL and 0.2% arabinose were inoculated into 200 µL of fresh media supplemented with 100 µg/mL of ampicillin and 0.2% of arabinose or glucose in a clear 96-well plate. The plate was covered with an optical film, with small holes poked at the side of each well to allow aeration. Incubation and OD measurements were performed with an Epoch 2 plate reader (BioTek) at appropriate temperatures with continuous shaking, and OD_600 nm_ was measured at 7.5 min intervals. Growth rate was calculated as the slope of ln(OD) with respect to time after smoothing using a moving average filter of window size five.

### Imaging acquisition on agarose pads

For fixed time-point experiments, cells were diluted 1:5,000 from an overnight culture. For experiments in Fig. 3A, B, 4B, C and 5E through G, diluted cells were grown for 3 h to an OD_600 nm_ ~0.1, then diluted 1:10 into fresh medium with appropriate inducers. For experiments in Fig. 6A, B, D and E; Fig. S4 and S5, diluted cells were grown to OD_600 nm_ = 0.4, then diluted 1:200 in 0, 0.25, 0.5, 1, 2, or 5 µg/mL A22. In these experiments, a small aliquot of cells (~1 µL) was placed onto agarose pads with M9+0.04% glucose every 30 min. Phase contrast and GFP fluorescence images were acquired as quickly as possible to avoid cell shape changes due to the medium change.

For time-lapse experiments, cells were diluted 1:5,000 from an overnight culture. For experiments in Fig. 3C, 4A and 5B through D, 3 h after the 1:5,000 dilution, cells were further diluted 1:10 and spotted onto M9 glucose pads (Fig. 3C), LB pads with 8 µM IPTG and 10 µg/mL cephalexin (Fig. 4A), or LB pads with 0.2% arabinose/glucose (Fig. 5B through D). For the experiment in Fig. 6C, at OD_600 nm_ = 0.2, cells were diluted 1:10 onto LB pads with 1% agarose and 0, 0.25, 0.5, 1, 2, or 5 µg/mL A22. Agarose pads were maintained at 37°C using a heated environmental chamber (Haison Tech). Phase contrast and fluorescence images were acquired every 2 min using a Nikon Ti-E epifluorescence microscope running µManager v. 1.4 (66).

### Imaging in microfluidic flow cells

For imaging experiments in Fig. 1C through H and Fig. 2, cells were diluted 1:500 from overnight cultures and grown for 3.5 h. Cells were then diluted to an OD_600 nm_ ~0.001 and introduced into a CellAsic flow cell pre-warmed to 37°C. Cells were then grown in LB for 20 min before switching to LB supplemented with IPTG.

### Single-cell measurements

All phase-contrast images were segmented using the Matlab-based software *Morphometrics* (27). For lineage tracking, cells were identified using the fully convolutional neural network model *DeepCell* (67). Outputs from *DeepCell* classification were used to extract cell contours with *Morphometrics*.

From the cell contours, a local coordinate system was generated using the pill-mesh algorithm from *MicrobeTracker* (68). Cell width was calculated as the average distance between contour points perpendicular to the cell midline along the cell body, and cell length was calculated as the length of the midline. Cell volume was estimated from width and length measurements by approximating the cell as a cylinder with hemispherical endcaps, such that $V=2\pi R^{2}\left(L-2R\right)+\frac{4}{3}\pi R^{3}$, where *R* is half the cell width and *L* is the cell length.

Single-cell growth rate was calculated using a time series of the cell volume estimated from the cell contours of a lineage. The instantaneous growth rate at time *t* was defined as $\frac{1}{V\left(t\right)}\frac{V\left(t+∆t\right)-V\left(t\right)}{∆t}$, where $V\left(t\right)$ is the cell volume at time *t*.

### Quantification of FtsZ fluorescence levels and spatial distribution

FtsZ rings were detected based on peaks in intensity along the contour. FtsZ ring intensity was calculated as the difference between the maximum and minimum of the fluorescence peak times the width of the peak, after background subtraction (45). Fluorescence intensity was calculated as the sum of all pixels in the fluorescence channel within the contour of the cell, divided by the cell volume, after background subtraction. For data in Fig. 4B, FtsZ ring distances were calculated based on adjacent fluorescence peaks as extracted from the midline of the cells.

### Western blot quantifications

After incubation with IPTG for at least 1.5 h, 1 mL of exponentially growing culture was harvested, lysed, and solubilized by boiling in Laemmli buffer for 5 min at 95°C. Samples were diluted and normalized based on OD_578 nm_ prior to SDS-PAGE analysis. Proteins were separated on a 15% SDS-PAGE gel and transferred to PVDF membranes (Immobilon-P). Membranes were blocked with 5% skim milk in TBS-T (50 mM Tris-HCl [pH 7.6], 0.15 M NaCl, and 0.1% Tween 20). TBS-T was used in all subsequent steps of the immunoblotting procedure. Anti-FtsZ (1:1,000, Acris) and anti-RecA (loading control, 1:1,000, Abcam) rabbit antisera were used as primary antibodies. Membranes were incubated with secondary antibodies conjugated with horseradish peroxidase diluted in 5% skim milk in TBS-T (1:10,000, GE Healthcare). Labeled proteins were detected via enhanced chemiluminescence (Pierce ECL Western Blotting Substrate, Thermo Scientific) and exposed on X-ray films (Kodak Biomax MR1).

The built-in gel analysis tool in *Fiji* (69) was used to quantify FtsZ and RecA levels from a horizontal rectangle, including the relevant bands.

## Data Availability

All raw data can be accessed at https://doi.org/10.25740/rv279br3622. Any other data is available upon request from the corresponding authors.

## SUPPLEMENTAL MATERIAL

Supplemental material mbio.02281-25-s0001.docx Supplemental figures and table.

ASM does not own the copyrights to Supplemental Material that may be linked to, or accessed through, an article. The authors have granted ASM a non-exclusive, world-wide license to publish the Supplemental Material files. Please contact the corresponding author directly for reuse.
