# Supplementary material for: Modulation of bacterial cell size and growth rate via activation of a cell envelope stress response
Source: mBio. 2025 Sep 22;16(11):e02281-25. doi: 10.1128/mbio.02281-25 (PMC12607864; doi:10.1128/mbio.02281-25)
Supplement: Supplemental material — Supplemental figures and table. [file mbio.02281-25-s0001.docx]

**Supplemental Information for** **“Modulation of bacterial cell size and growth rate via activation of a cell envelope stress response”**

**Authors:** Amanda Miguel^1,*^, Matylda Zietek^2,*^, Handuo Shi^1,3,*^, Anna Sueki^2,4^, Federico Corona^2^, Lisa Maier^2^, Jolanda Verheul^5^, Tanneke den Blaauwen^5^, David Van Valen^1,6^, Athanasios Typas^2,7,†^, Kerwyn Casey Huang^1,3,8,†^

^1^Department of Bioengineering, Stanford University, Stanford, CA 94305, USA

^2^Genome Biology Unit, EMBL Heidelberg, Meyerhofstraße 1, 69117 Heidelberg, Germany

^3^Department of Microbiology and Immunology, Stanford University School of Medicine, Stanford, CA 94305, USA

^4^Collaboration for joint PhD degree between EMBL and Heidelberg University, Faculty of Biosciences, Germany

^5^Faculty of Natural Sciences, Mathematics, and Computer Science, Swammerdam Institute for Life Sciences, University of Amsterdam, The Netherlands

^6^Department of Biology, California Institute of Technology, Pasadena, CA 91125, USA

^7^Molecular Systems Biology Unit, EMBL Heidelberg, Meyerhofstraße 1, 69117 Heidelberg, Germany

^8^Chan Zuckerberg Biohub, San Francisco, CA 94158

Short title: *Rcs activation reduces size*, *growth rate*

^*^These authors contributed equally.

^†^Correspondence: [typas@embl.de](mailto:typas@embl.de) and [kchuang@stanford.edu](mailto:kchuang@stanford.edu)

**Supplemental Figures**

**
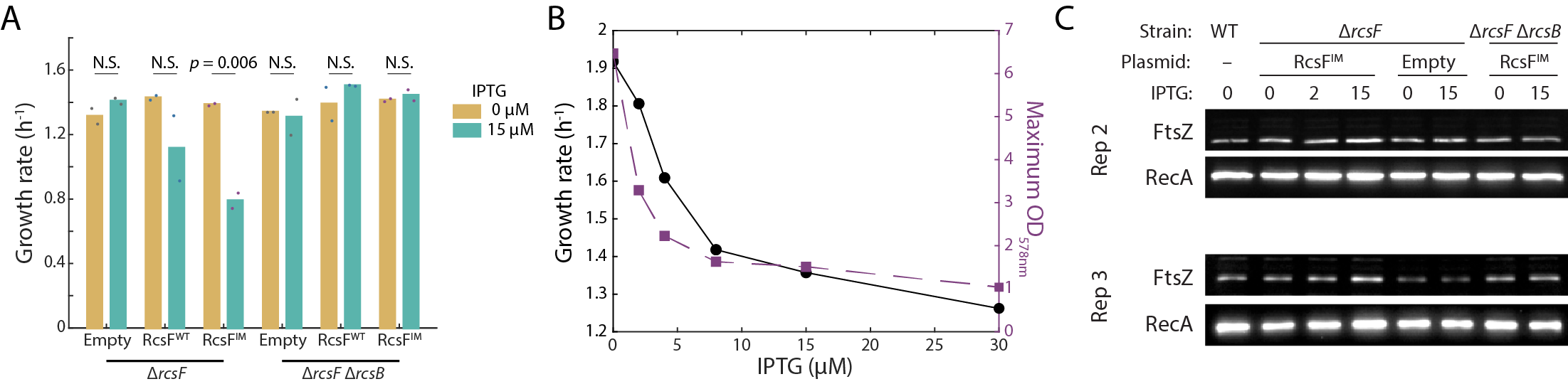
**

**Figure S1: RcsF^IM^ induction slows growth and is dependent on *rcsB*.**

1. The growth rate decrease caused by IPTG induction of RcsF^IM^ production was dependent on the presence of *rcsB*. *n*=2 replicates for each condition. *p*-values are from two-tailed Student’s *t*-tests. N.S.: not significant.
2. RcsF^IM^ induction led to a decrease in steady-state growth rate and stationary-phase yield in batch culture. Yield was measured after 8 h of growth from the initial dilution; all growth curves plateaued by that time. Data are from *n*=1 replicate.
3. Replicates of Western blotting as in Fig. 3E. In all replicates, the ratio of FtsZ to RecA levels increased dependent on IPTG concentration in RcsF^IM^ cells but not in cells with an empty vector or cells lacking *rcsB*.

**
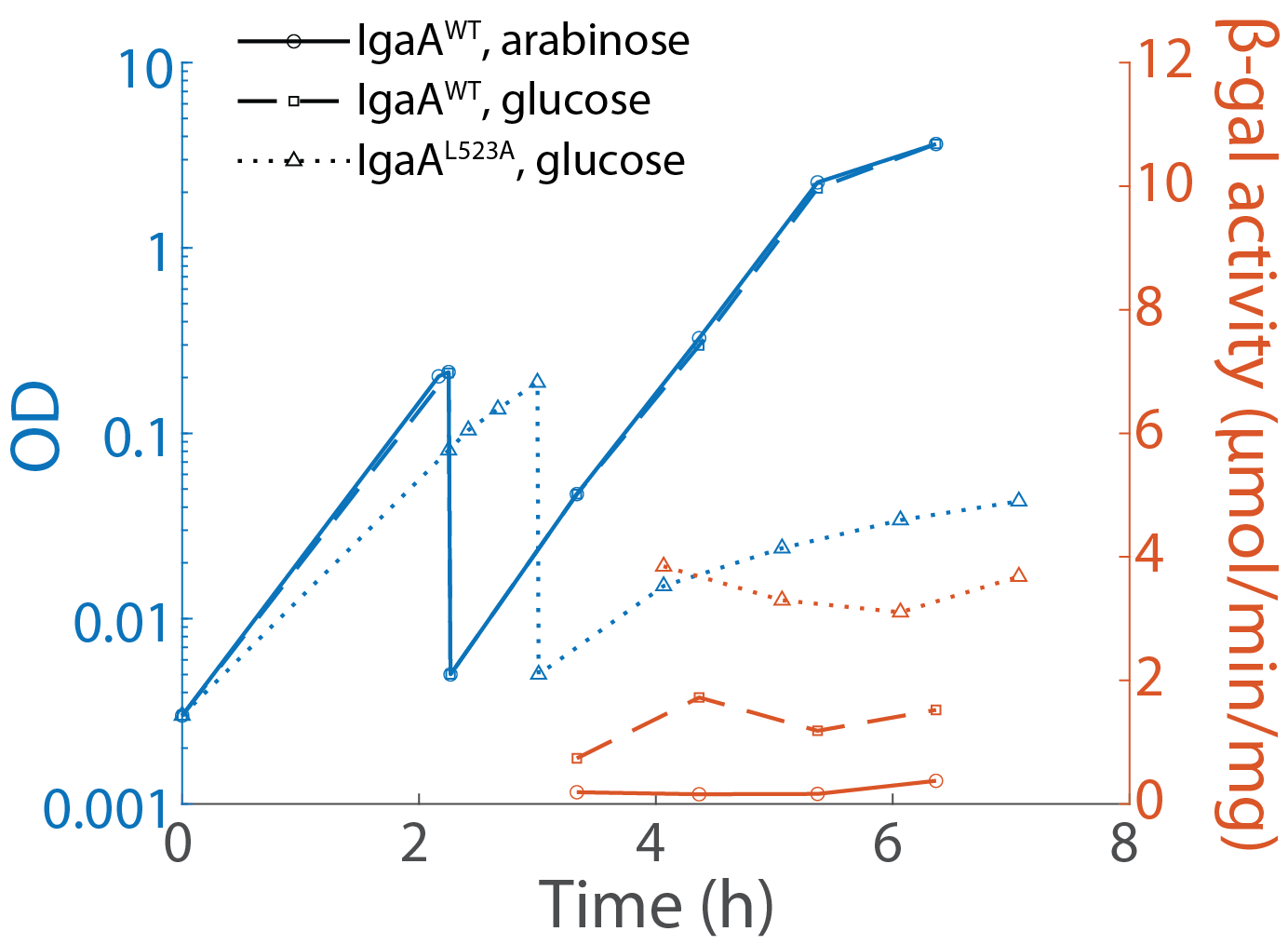
**

**Figure S2: Depletion of IgaA^L523A^ decreases growth rate and activates the Rcs system.** Activation was measured through induction of chromosomal *rprA::lacZ* in a β-galactosidase assay (right). Cells were switched to glucose at *t*=0. Depletion of IgaA^WT^ only mildly activated Rcs expression and did not affect cell growth over the time scale of measurement. In contrast, depletion of IgaA^L523A^ reduced growth rate and exhibited higher Rcs activation. Cultures were diluted 1:100 after ~2-3 h. Data are from *n*=3 replicates.

**
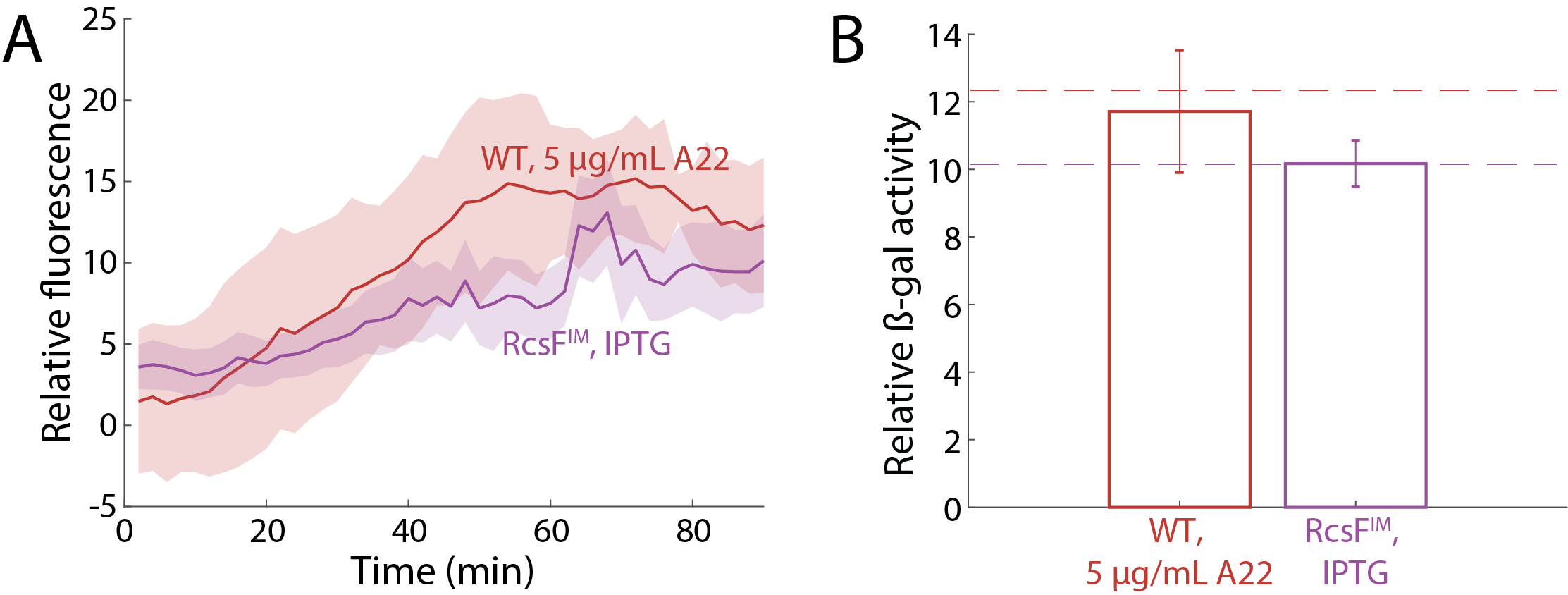
**

**Figure S3: RcsF^IM^ induces activation of the Rcs pathway to a similar degree as A22 treatment.**

1. RcsF^IM^ induces the Rcs pathway with similar dynamics as A22 treatment, as quantified using msfGFP fluorescence from the *rprA* promoter. Wild-type (WT) fluorescence is relative to the control without A22 treatment, and RcsF^IM^ fluorescence is relative to ∆*rcsF* cells with an empty vector. Curves are medians and shaded regions represent 1 SD, estimated from the median absolute deviation. *n*>30 cells for every time point.
2. RcsF^IM^ induces the Rcs pathway to a similar degree as A22 treatment, as quantified by relative beta-galactosidase activity of the *rprA* promoter. *n*=4 for WT and *n*=3 for RcsF^IM^, and data points are mean±1 SD. Dashed lines represent the promoter activities measured by msfGFP fluorescence in (A) at *t*=90 min.

**
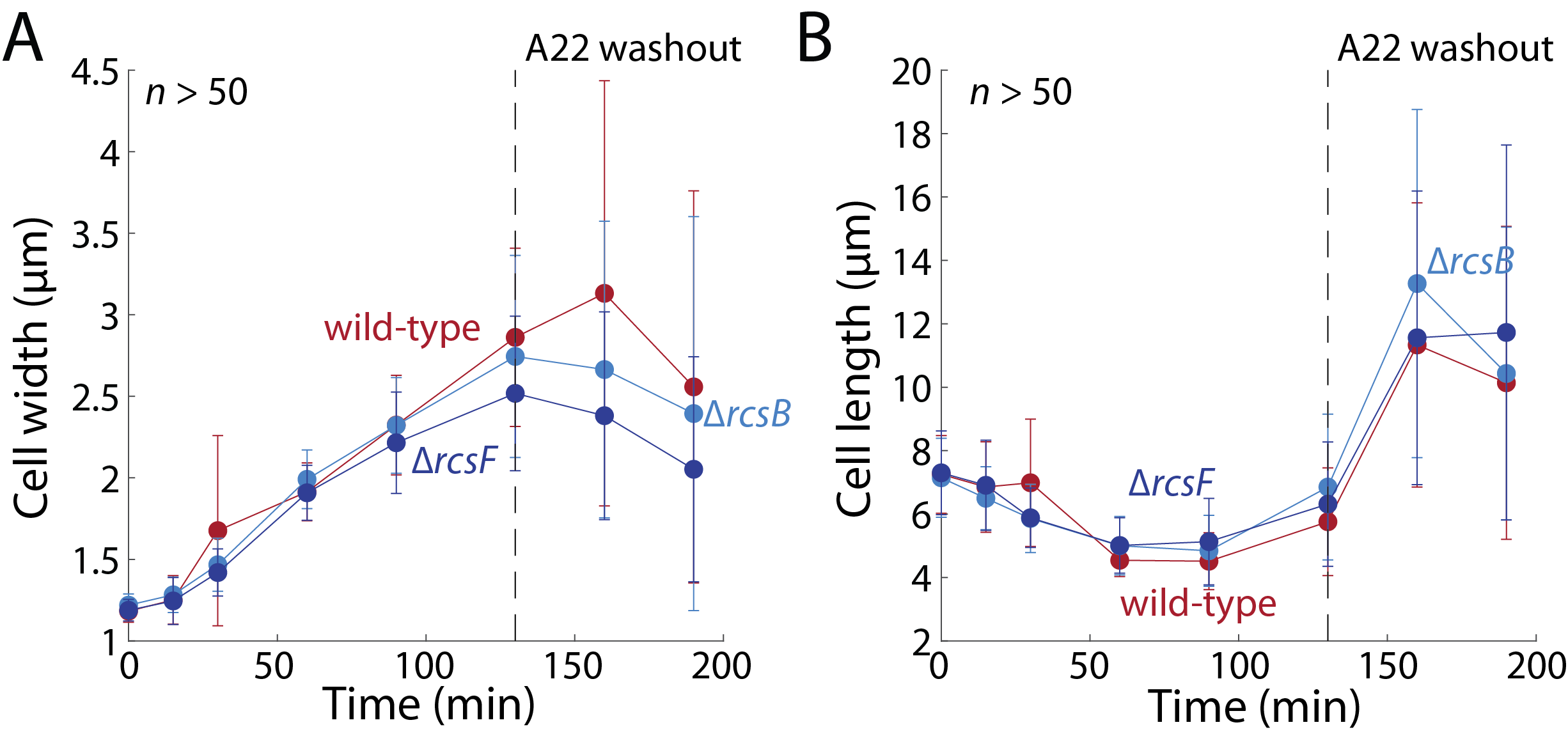
**

**Figure S4: Cell shape recovery after A22 treatment is Rcs-independent.**

Cells growing in LB were treated with 5 µg/mL A22 at *t_­_*=0 and aliquots of the cultures were sampled periodically to measure the dynamics of width (A) and length (B). After 130 min, cells were resuspended in LB without A22. Similar dynamics were observed in wild-type, ∆*rcsF*, and ∆*rcsB* cells. Data points are mean±1 standard deviation (SD) with *n*>50 cells.

**
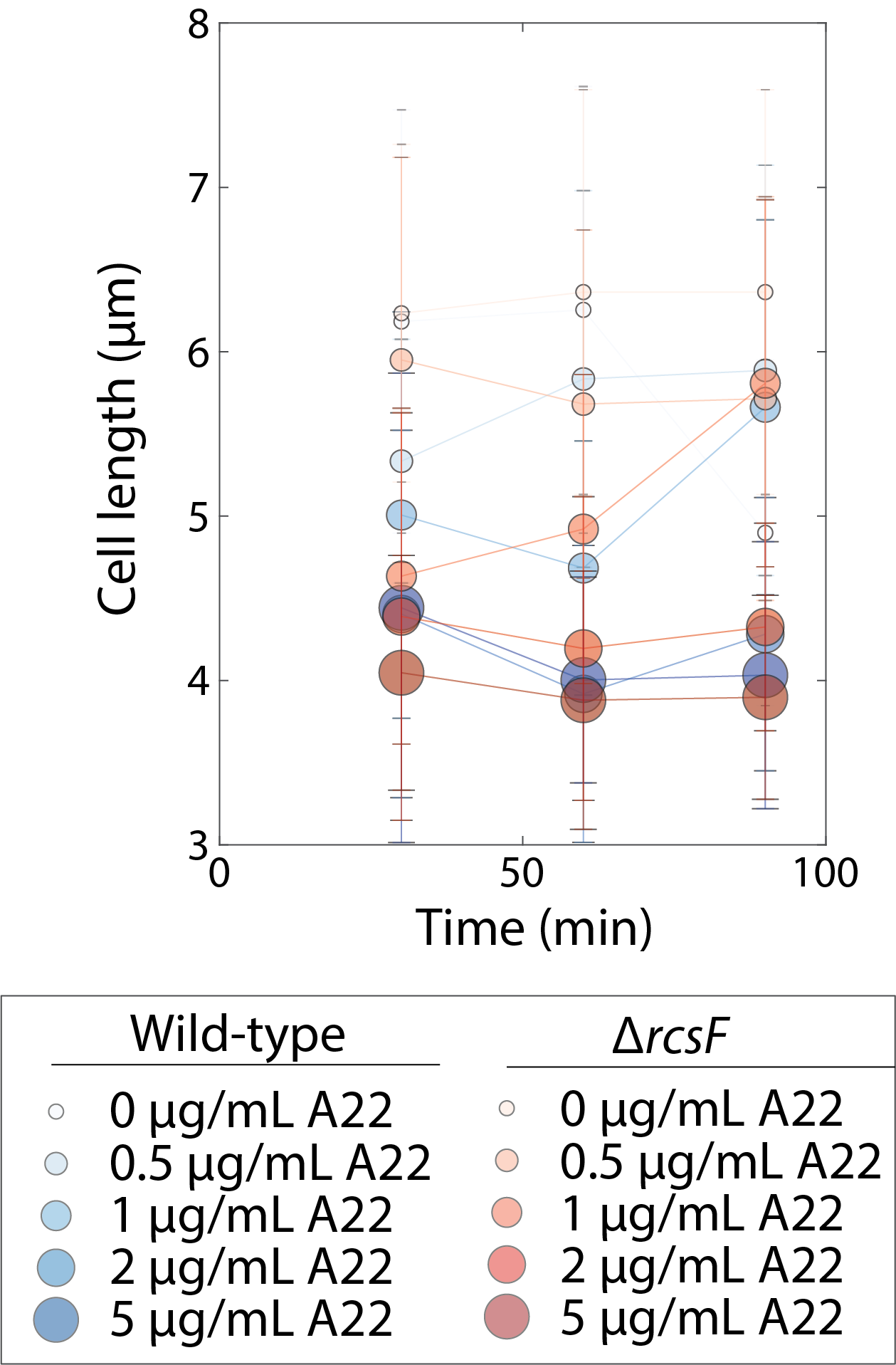
**

**Figure S5: Cell length decreases in an A22 dose-dependent but RcsF-independent manner.** Cells analyzed are the same ones as in Fig. 6. Data are mean±1 SD with *n*>50 cells.**Supplemental Tables**

**Table S1: Strains and plasmids used in this study.**

| **Resource** | **Reference or source** | **Identifier or catalog number** |
| --- | --- | --- |
|  |  |  |
| **Strains** |  |  |
| *E. coli rprA*::*lacZ* MG1655 (*argF-lac*)*U169* (used as wild type) | (41) | DH300 |
| *E. coli* DH300 Δ*rcsF::kan* | This study | NT15048 |
| *E. coli* DH300 Δ*rcsF::cat* Δ*wcaJ::kan* | This study | AM110 |
| *E. coli* DH300 Δ*rcsF::kan* Δ*rcsB::cm* | This study | NT15178 |
| *E. coli* DH300 Δ*rcsF ftsZ*::*ftsZ-msfGFP* | (44), this study | AM115 |
| *E. coli* DH300 pBAD30-*igaA^L523A^* Δ*igaA::cat* | This study | KC1183 |
| *E. coli* DH300 Δ*rcsF::kan* pBAD30-*igaA^L523A^* Δ*igaA::cat* | This study | KC1185 |
| *E. coli* DH300 pBAD30-*igaA^L523A^* Δ*igaA::cat* *ftsZ*::*ftsZ-msfGFP* | This study | AM181 |
| **Plasmids** |  |  |
| pAC581 | (70) |  |
| pBAD30- IgaA^L523A^ | This study | IgaA^L523A^ under arabinose-induced promoter, ampicillin resistance |
| pMZ13 | This study | msfGFP under *rprA* promoter, pAC581-based, chloramphenicol resistance |
| pNG162-Empty | (8) | Control plasmid, spectinomycin resistance |
| pNG162-RcsF-WT | (8) | RcsF^WT^ under IPTG-induced promoter, spectinomycin resistance |
| pNG162-RcsF-IM | This study | RcsF^IM^ under IPTG-induced promoter, spectinomycin resistance |
| pTrcHis2A | Invitrogen | Ampicillin resistance |
